# High Accuracy Measurement of Aperture Area Relative to a Standard Known Aperture

**DOI:** 10.6028/jres.100.020

**Published:** 1995

**Authors:** Joel B. Fowler, Gyula Dezsi

**Affiliations:** National Institute of Standards and Technology, Gaithersburg, MD 20899-0001; National Office of Measures, Budapest, Hungary

**Keywords:** aperture, area, measurement, relative

## Abstract

Precise knowledge of the area of apertures used in high precision radiometry is extremely important. A method is presented here for the determination of the area of round and irregularly shaped apertures by comparison to a standard aperture which has been measured by other means to high accuracy. The method presented here is quick and has no physical contact with the fragile edge of the aperture opening. Total flux transfer methods are used in the area determination with total relative standard uncertainty of 0.033 % for 2 mm to 25 mm mean diameter apertures not including the area uncertainty of the standard aperture used. Currently the relative standard uncertainty in the area measurement for the stadard aperture is 0.022 %. The worst case relative standard uncertainty of the transfer measurement is 0.04 % including the uncertainty of the standard aperture area.

## 1. Introduction

As the total uncertainty in radiometric measurements has decreased over the last 10 years, the accurate knowledge of the area of the limiting aperture on sources and detectors has become increasingly important. In the 1990s measurements using absolute cryogenic radiometers and other high accuracy radiometric instruments with very low measurement uncertainties require knowledge of the area of the apertures used in conjunction with these instruments to also have very low area uncertainties [1, 2]. Methods generally used to measure aperture area to high accuracy have been of two types, contact and optical noncontact. The contact method requires the use of a stylus to mechanically sense the fragile edge around the opening in the aperture. This requires the land (the sharp interior edge of the aperture) to be large, resulting in unwanted scatter and reflections from the land surface when used for optical measurements. If the land is narrow, the stylus deforms the sharp land edge resulting in decreased accuracy of the measurement. Both the contact method and the optical methods generally entail the measurement of large numbers of diameters if the aperture geometry is not adequately known.

A new and novel technique is used at the Physikalisch-Technische Bundesanstalt (PTB) in Germany which employs a laser and detector in a configuration that eliminates many of the objections to optical area measurements, resulting in low uncertainty of the overall measurement. Large numbers of diameter determinations still must be made to account for the geometry of the aperture opening, but since the uncertainty of the basic method is very low, the total uncertainty of this method is also very low for many apertures.

The instrument described here provides a means to measure the area of a round or *irregularly* shaped aperture in the range of 2 mm to 25 mm diameter relative to the area of an aperture of known area with a combined standard relative uncertainty of 0.033 % plus the uncertainty of the standard aperture (all uncertainties stated in this paper are 1*σ* uncertainties, that is, one standard uncertainty). This method requires only one direct determination of the area of the unknown aperture relative to the standard aperture. Current plans include an effort to decrease the total relative standard uncertainty of the instrument to less than 0.01 % including the relative standard uncertainty in the area of the standard aperture.

## 2. Approach

**Fig. 1:**
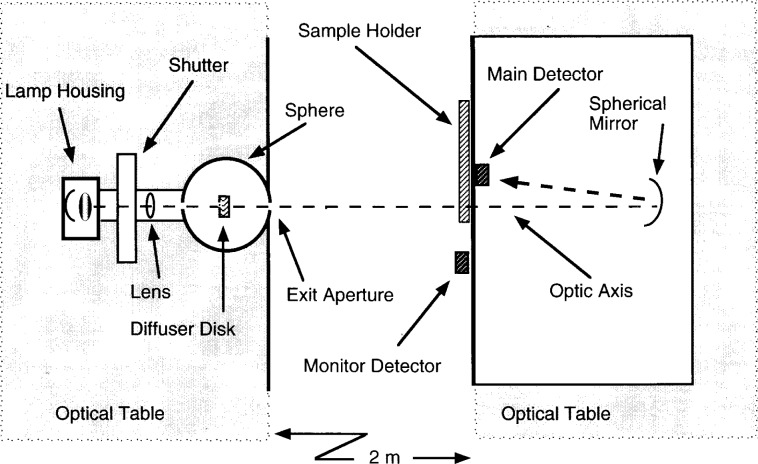
System layout.

The instrument shown in Fig. 1 is a flux comparator consisting of a sphere source illuminating either the standard aperture or the unknown aperture at a sufficient distance to provide uniform irradiance (*E_λ_*) across the aperture. The flux through the selected aperture is collected by a spherical mirror and the image of the sphere source exit aperture is focused onto a windowless Hamamatsu 1337-1010 silicon detector.^1^ The photocurrent is amplified, converted to a voltage, and measured with a high accuracy digital voltmeter. The lamp flux is monitored by a second Hamamatsu silicon detector of the same type, located adjacent to the aperture test position and measured by a second digital voltmeter.

## 3. Aperture Test Apparatus

As shown in Fig. 1, the sphere source is positioned on an optical table on the left. The nominal sphere diameter is 100 mm with two ports on opposite sides and a diffuser disk in the center. The diffuser disk is nominally 14 mm thick × 25 mm in diameter, located at the center of the sphere, on axis with the input and output ports, and much larger than either the incoming beam diameter or the output field of view. The disk is fabricated from sintered teflon of sufficient thickness to block any direct transmission of the incoming light and is held in place with a small metal rod coated with sintered teflon. The input port is nominally 8 mm in diameter and the exit aperture is a thin 8 mm diameter beryllium-copper aperture applied over the exit port. Although the exact diameter of the exit aperture is unimportant, it’s area must be constant or taken into account for measurements made at very low uncertainties. The beryllium-copper material used for this aperture, along with the known temperature stability of the room containing the instrument, fulfills the area stability requirement at the present level of uncertainty.

The optical flux is produced by a 100 W quartz halogen lamp housed in a commercial enclosure which utilizes a spherical re-imaging mirror to increase the usable flux. The output port of the lamp housing is fitted with an electronic shutter which permits background readings to be recorded. The radiation from the lamp is defined by a stable aperture which is then focused at the input port of the sphere using a 50 mm diameter, 127 mm focal length lens at a suitable location. The radiation path from the lamp housing to the sphere entrance is enclosed by a round 75 mm diameter tube painted flat black inside. The lens is situated inside this tube as shown in Fig. 1. Nearly all of the radiation passing through the tube is collected on the diffuser disk inside the sphere eliminating any direct reflections to the exit aperture.

The standard and unknown apertures are held in a 30.5 cm diameter circular sample holder with 50 mm diameter holes spaced every 45° just inside the circumference. The sample holder is rotated by a micro stepping motor system which is computer controlled. Each aperture is aligned by concentric circular steps in the surface at each position along with special mounting rings where needed. The apertures are positioned in the mounting rings so as to maintain a uniform position in the plane normal to and concentric with the axis of the beam path.

The sample holder is positioned at the front of a light tight box located 2 m from the exit port of the sphere and situated on a second optical table. The box contains a 0.5 m radius, 10 cm diameter spherical mirror and a silicon detector. The sphere exit aperture, demagnified to approximately 1 mm diameter, is imaged on the main detector. Care was taken in the positioning of the internal components to minimize light scattering problems and a strip of black cloth is positioned to absorb the reflection from the surface of the silicon detector.

A 1.2 m × 1.2 m aluminum plate covered with diffuse black cloth is positioned in front of the exit port of the sphere. The cloth absorbs any reflections from the table and other objects which could scatter or reflect into the box through the aperture currently positioned at the test location. The sphere output port extends slightly through a tapered hole in the plate. The plate entirely fills the field of view of the test apparatus. The entire beam path at the 1.2 m boundaries of the plate is enclosed by black cloth to exclude any residual stray light entry from the already dark room.

The optical components are aligned along the optical axis using a HeNe alignment laser. Angular alignment is checked and adjusted using a small flat mirror applied first to the test aperture holder and then to the sphere source aperture holder, aligning each component until the beam is reflected back along the optical axis to the laser. The alignment of the optical axis centerline was adjusted by attaching two apertures the same diameter as the HeNe laser beam, one to the test aperture location and one to the sphere source output port. The sphere source and the sample holder are re-positioned until the HeNe laser beam passes through the center of each aperture. The beam entering the box is centered on the collecting mirror using a paper mask with an “x” in the center positioned just in front of the mirror surface. The converging beam from the mirror is aligned on the approximate center of the location of best uniform responsivity on the surface of the silicon detector and focussed by moving the detector along the beam axis until there is no lateral movement of the beam when the aperture in the sample holder is rotated through the beam path. The silicon detector is turned at a slight angle and the reflection absorbed on the piece of black cloth behind the collecting mirror.

Intensity uniformity measurements were made at the test position in the plane of the sample holder at a distance of 2 m from the sphere exit aperture. An *x*–*y* positioner was used to scan the central 50 mm diameter of the beam at 1 mm intervals with a silicon detector fitted with a 2 mm diameter aperture. The signal from the detector was recorded in a 50 point × 50 point matrix. The maximum intensity deviation normalized to the center of the beam was 0.01 %. The *x–y* positioner was used to scan the same central 50 mm diameter of the beam at the same positions using only the 2 mm diameter aperture, the flux now being collected by the spherical mirror and measured by the main detector. The voltage recorded from the main detector is then a measure of the combination of the error due to mirror reflectance nonuniformity and sphere source flux non-uniformity as a single quantity. The maximum deviation of the beam intensity thus measured never exceeded 0.018 % relative to the center of the beam. These measurements indicate a significant contribution from both the source flux nonuniformity and the mirror reflectance nonuniformity. The standard uncertainty for the mirror nonuniformity and the sphere nonuniformity as measured will be used as a single quantity in later analysis.

Small diameter apertures produce diffraction patterns which must be collected and transferred to the detector. From studies and calculations done at the present level of uncertainty, the 100 mm diameter of the spherical mirror is large enough to collect a sufficient number of these diffraction rings, so as to introduce no significant error with respect to the uncertainties in the measurement.

## 4. Measurement Details

The standard aperture and the test aperture are irradiated with the flux from the sphere source which is intensity monitored with a silicon detector located at a position adjacent to and 25 mm to the right of the aperture test location. The aperture of interest is located in the sample holder 2 m from the sphere source. This location was chosen to ensure that the beam sufficiently approximates a point source while maximizing the optical throughput and producing a small spot size at the main detector.

The spot size at the detector is important because of the nonuniformity of response of silicon detectors. The detector used was carefully selected for uniformity of response at 1000 nm. Although the detector still had relatively large deviations over much of its surface, a 4 mm square area was found which exhibited a small response nonuniformity. The beam is centered in an area within this square with a response nonuniformity of approximately 0.01 %. If the beam is carefully focused at this spot, so that it does not perceptibly move as the aperture in the sample holder is rotated through the beam, the effects from response nonuniformity are minimized to an insignificant amount.

The shutter-closed background is measured. Next the flux through the standard aperture is measured simultaneously with the lamp intensity monitor. Ten measurements are made of these two quantities. The sample holder is rotated to a position containing an unknown aperture and another ten measurements of the flux is made along with the lamp intensity monitor and the associated background. The mean and standard deviation of the mean of these measurements is calculated and the area of the test aperture determined from the results.

## 5. Measurement Equation

It will be shown in this section that the area of the unknown aperture *A*_test_ is approximately equal to the product of the area of the standard aperture *A_std_* and the ratio of the voltages produced due to the flux through the unknown aperture *V*_test_ and the voltage produced by the flux through the standard aperture *V*_std_ as follows:

$$A_{\text{test}}\approx A_{\text{std}}\cdot \frac{V_{\text{test}}}{V_{\text{std}}}.$$ (1)

Equation (1) assumes the irradiance field is uniform and the responsivity of the detectors are constant in time, and linear with respect to the incident power level used here.

The complete measurement equation is derived by considering that the voltage *V* produced by a silicon detector as used here is directly proportional to the area *A* of the aperture in front of the detector, the irradiance *E_λ_*, and the responsivity *R* of the detector:

$$V=A\cdot E_{\lambda }\cdot R.$$ (2)

Equation (2) assumes that *R* is independent of *E_λ_*, and the diodes used are known to be highly linear with respect to response. In the procedure described here, two separate detectors are used: the monitor detector and the main detector. The monitor detector is used to correct temporal instabilities in the light source during the course of the experiment. The normalization of the voltages with respect to the monitor detector results in four separate voltage determinations given by Eq. (2) to determine the unknown aperture area. Additionally, each voltage measurement is referred to the zero determined by a background measurement with the shutter closed.

The two voltages *V*_test_ and *V*_std_ are normalized to their respective monitor voltages $V_{{\text{mon}}_{\text{test}}}$ and $V_{{\text{mon}}_{\text{std}}}$ accounting for any temporal drift of the light source. Background readings are subtracted and ratios applied to account for nonuniform irradiance fields as shown below:

$$A_{\text{test}}=A_{\text{std}}\cdot \frac{V_{\text{test}}-VBG_{\text{test}}}{V_{{\text{mon}}_{\text{test}}}-VBG_{{\text{mon}}_{\text{test}}}}\cdot \frac{V_{{\text{mon}}_{\text{std}}}-VBG_{{\text{mon}}_{\text{std}}}}{V_{\text{std}}-VBG_{\text{std}}}\cdot \frac{E_{\lambda }^{{\text{mon}}_{\text{std}}}}{E_{\lambda }^{\text{std}}}\cdot \frac{E_{\lambda }^{\text{test}}}{E_{\lambda }^{{\text{mon}}_{\text{test}}}},$$ (3)

where *VBG*_test_ is the shutter closed background voltage from the main detector amplifier with the unknown aperture in the optical path, *VBG*_std_ is the shutter closed background voltage from the main detector amplifier with the standard aperture in the optical path, $VBG_{{\text{mon}}_{\text{test}}}$ is the shutter closed background voltage from the monitor detector amplifier with the unknown aperture in the optical path, $VBG_{{\text{mon}}_{\text{std}}}$is the shutter closed background voltage from the monitor detector amplifier with the standard aperture in the optical path, *V*_test_ is the voltage from the main detector amplifier due to the flux through the unknown aperture, *V*_std_ is the voltage from the main detector amplifier due to the flux through the standard aperture, $V_{{\text{mon}}_{\text{test}}}$ is the voltage from the monitor detector amplifier due to the flux from the sphere source during the measurement of the unknown aperture and $V_{{\text{mon}}_{\text{std}}}$ is the voltage from the monitor detector amplifier due to the flux from the sphere source during the measurement of the standard aperture, $E_{\lambda }^{\text{std}}$ is the irradiance at the test position when the standard aperture is being measured, $E_{\lambda }^{\text{test}}$ is the irradiance at the test position when the unknown aperture is being measured, $E_{\lambda }^{{\text{mon}}_{\text{test}}}$ in the irradiance at the monitor detector when the unknown aperture is being measured, $E_{\lambda }^{{\text{mon}}_{\text{std}}}$ and is the irradiance at the monitor detector when the standard aperture is being measured. The drift in photodiode responsivity is insignificant as silicon detectors are known to have a responsivity that is stable to better than 0.001 % in the time scale and the conditions of this measurement, and hence the ratio of the responsivities of the detectors cancel out of the equation. The monitor detector is overfilled and is assumed to be unaffected by spatial non-uniformity of the detector.

The measurement is accomplished using two digital voltmeters triggered simultaneously to measure the signal and the monitor at the same moment correlating them in time. The statistical uncertainty of each of these ratios may be determined by calculation of the standard deviation of the mean of a number of measurements of each of the quantities

$$\frac{V_{\text{test}}-VBG_{\text{test}}}{V_{{\text{mon}}_{\text{test}}}-VBG_{{\text{mon}}_{\text{test}}}}$$ (4)

and

$$\frac{V_{{\text{mon}}_{\text{std}}}-VBG_{{\text{mon}}_{\text{std}}}}{V_{\text{std}}-VBG_{\text{std}}};$$ (5)

the results are then used along with the irradiance ratios to calculate the area of the unknown aperture. The ratio of the irradiance at the aperture holder to the irradiance at the monitor detector is assumed to be constant and independent of temporal fluctuations in the source. This assumption is generally valid to high accuracy for a Lambertian source in the far field. Thus the value of the ratios for the test and standard apertures in Eq. (3) is assumed to be unity. In addition to correcting for the drift in the source intensity, the standard deviation of the ratios in Eqs. (4) and Eq. (5) are reduced by integrating the signals from the monitor and main detectors over the same period of time starting at the same instant. This is important as the source fluctuations are typically 0.1 % on the time scale of a single measurement.

Each measurement sequence consists of 10 data points for the standard aperture and 10 data points for each test aperture. The mean of these measurements is then used in the calculation of the area of the unknown aperture.

## 6. Measurement Uncertainties

Two major contributors to the combined standard uncertainty of the measurement are the uncertainties associated with the area of the standard aperture, and the nonuniformity of the reflectance over the surface of the spherical mirror combined with the nonuniformity of the irradiance from the sphere source.

The standard aperture currently in use has an area of 452.48 mm^2^ and contributes a relative standard uncertainty of 0.022 %, as measured by the dimensional metrology group at NIST Gaithersburg. The sphere source flux and mirror reflectance contribute a relative standard uncertainty of 0.018 % when measured as a unit. The sphere source, when measured separately, contributes a relative standard uncertainty of 0.01 % due to spatial nonuniformity of the beam. The mirror then contributes a relative standard uncertainty of approximately 0.01 % due principally to the variability of the reflectance over the surface.

The temperature in the area at the test location is monitored in real time with an environmental monitor with a 0.1 °C standard uncertainty. The temperature in this area is stable to better than 1 °C over a period of 8 h and better than 0.2 °C over the period of the measurement. The computer program monitors the temperature and if it changes by more than 0.2 °C during the course of the measurement, a flag is set to alert the operator. This temperature is sufficiently stable to contribute a relative standard uncertainty of less than 0.001 % and can be ignored. Corrections to the areas of both the unknown and standard apertures are applied to the final determination to account for actual room temperature at the time of the measurement. The final area is adjusted to 20 °C for the purposes of uniformity in specification. Additional uncertainties in the measurement are due to uncertainties from the equipment used. These uncertainties are part of the uncertainty of the measurement of the flux and will not be addressed separately.

The relative standard uncertainties used in the calculation of the relative combined standard uncertainty are listed in Table 1. The relative standard uncertainties are added in quadrature to calculate the relative combined standard uncertainty:

$$u_{c,r}\left(A_{\text{test}}\right)=\sqrt{u_{r}^{2}\left(V_{\text{test}}\right)+u_{r}^{2}\left(V_{\text{std}}\right)+u_{r}^{2}\left(V_{{\text{mon}}_{\text{test}}}\right)+u_{r}^{2}\left(V_{{\text{mon}}_{\text{std}}}\right)}+\sqrt{u_{r}^{2}\left(E_{\lambda }^{\text{test}}\right)+u_{r}^{2}\left(E_{\lambda }^{\text{std}}\right)+u_{r}^{2}\left(A_{\text{std}}\right)}.$$ (6)

The uncertainties of the background for the monitor and test locations are small compared to the relative combined standard uncertainty and ignored.

## 7. Results

The greatest relative combined standard uncertainty for the measurement of the area of the unknown aperture is 0.04 % for apertures in the range of 2 mm to 25 mm mean diameter. The above measurement sequences were repeated 10 times for each unknown aperture. Ten additional area determinations were then calculated for each of the unknown apertures showing less than 0.015 % deviation from the original calculated area for that particular aperture. This indicates a minimum stability of 0.015 % in the basic measurement.

Representative areas and uncertainties are presented in Table 2 along with the area and uncertainties as determined by means other than this apparatus. Comparing the areas and uncertainties determined using this apparatus to the areas and uncertainties determined by the other means, it is apparent that the areas determined with the described apparatus differ from the areas measured by other means, but always within the uncertainty of the respective measurement.

## 8. Conclusions

With the available equipment and standard apertures this measurement scheme shows great promise of attaining our ultimate goal of 0.01 % or better relative combined standard uncertainty. We are currently modifying this instrument by improving the sphere source, obtaining a new spherical mirror, and mounting the entire apparatus in a sealed light-tight box to eliminate stray light. We are fabricating new standard apertures machined using state of the art diamond turning techniques. The uniformity of the reflectance of the spherical mirror currently used is being improved by employing a state-of-the-art high reflectivity coating on a highly polished mirror blank. The improved instrument will be the subject of a future publication.

## Figures and Tables

**Table 1:** Relative standard uncertainty components^a^

Source of uncertainty	Relative standard uncertainty (%)
Voltage from the main detector amplifier, *V*_test_	0.014
Voltage from the main detector amplifier, *V*_std_	0.014
Voltage from the monitor detector amplifier, $V_{{\text{mon}}_{\text{test}}}$	0.014
Voltage from the monitor detector amplifier, $V_{{\text{mon}}_{\text{std}}}$	0.014
Irradiance at the test plane during meas. of unknown aperture, *E_λ_*^test^	0.018
Irradiance at the test plane during meas. of standard aperture, $E_{\lambda }^{\text{std}}$	0.018
Standard aperture area, *A*_std_	0.022
Mirror reflectance nonuniformity(b)	0.01
Sphere source aperture area (% per degree Celsius)(b)	0.0034
Corrected lamp Drift(b)	<<0.01
DVM uncertainty(b)	0.005
DVM linearity(b)	0.0035
Amplifier drift (% per dgree Celsius)(b)	0.003
Relative combined standard uncertainty of the measurement, *u*_c,r_(*A*_test_)	0.04

a All uncertainties are type A.

b Indicates a quantity which was measured for completeness but not included in the uncertainty calculations.

**Table 2:** Representative experimental results

Aperture designation	Measured area (mm^2^) (this work)	Relative standard uncert. (%)	Measured area (mm^2^) (other)	Relative Standard uncert. (%)	Source^a^	Material
PA1	13.23	0.035	13.14	0.15	NIST S	BeCu
PA2	13.01	0.035	12.89	0.34	NIST S	BeCu
PA3	12.747	0.035	12.62	0.21	NIST S	BeCu
PA4	15.06	0.035	NA(b)	NA(b)	NIST S	BeCu
PA5	13.608	0.035	13.46	0.19	NIST S	BeCu
PA6	12.942	0.035	12.942	0.02 (3)	PTB	BeCu
PA7	28.928	0.035	28.7	0.18	NIST S	BeCu
PA8	28.948	0.029	28.8	1.45	NIST S	BeCu
X1	49.96	0.039	49.96	0.063	NIST P	BeCu
Z1	49.917	0.038	49.95	0.063	NIST P	BeCu
A1	346.45	0.029	346.44	0.024	NIST P	Al
A4(8)	9.985	0.039	10.01	0.14	NIST P	BeCu
A6	10.001	0.035	10	0.11	NIST P	BeCu

a NIST S = NIST Shops, PTB Physikalisch-Technische Bundesanstalt, NIST P = NIST Precision Metrology Group. All measurements done by others are of the optical noncontact type.

b Not Available

## Footnotes

1 Certain commercial equipment, instruments, or materials are identified in this paper to foster understanding. Such identification does not imply recommendation or endorsement by the National Institute of Standards and Technology, nor does it imply that the materials or equipment identified are necessarily the best available for the purpose.
